# Supplementary material for: Biodistribution of Intratracheal, Intranasal, and Intravenous Injections of Human Mesenchymal Stromal Cell-Derived Extracellular Vesicles in a Mouse Model for Drug Delivery Studies
Source: Pharmaceutics. 2023 Feb 7;15(2):548. doi: 10.3390/pharmaceutics15020548 (PMC9964290; doi:10.3390/pharmaceutics15020548)
Supplement: Supplementary file 1 [file pharmaceutics-15-00548-s001.zip › pharmaceutics-2111910-supplementary.pdf]

SUPPLEMENTARY FIGURES

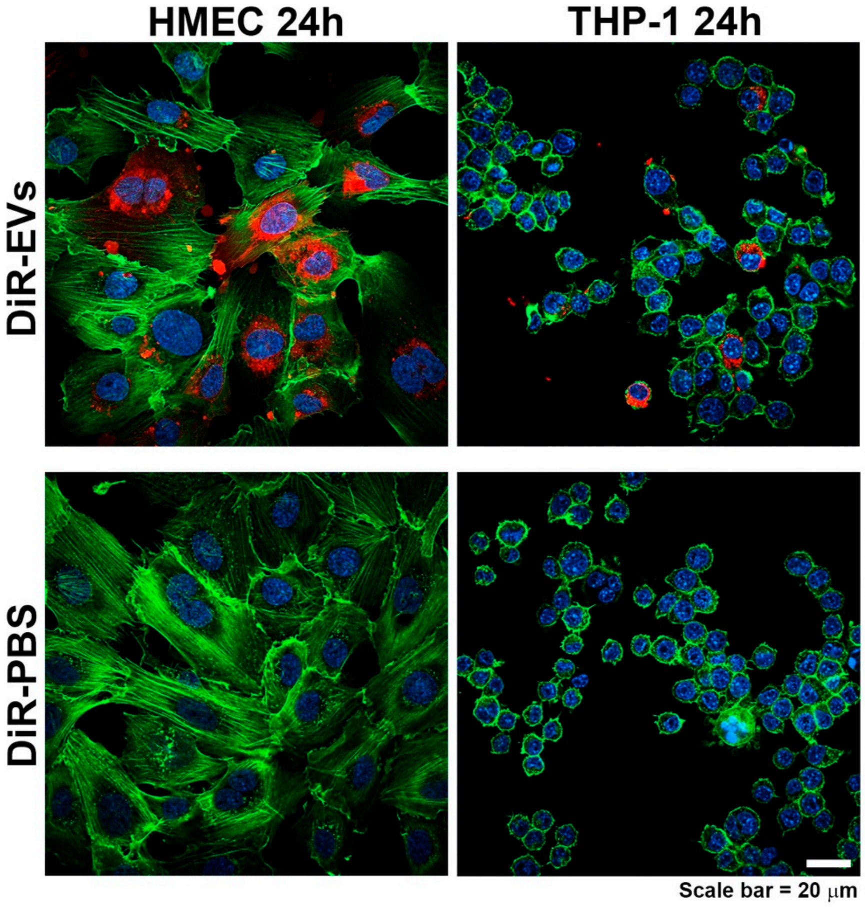

Figure S1: In-vitro uptake experiments. HMEC and THP-1 were incubated in the presence of DiR-labeled PBS (DiR-PBS) or of DiR-labeled EVs (DiR-EVs) for 24 hours (in red). Cells are labeled in FITC-phalloidin (green) for cytoskeleton staining and DAPI (blue) for the nuclear staining.

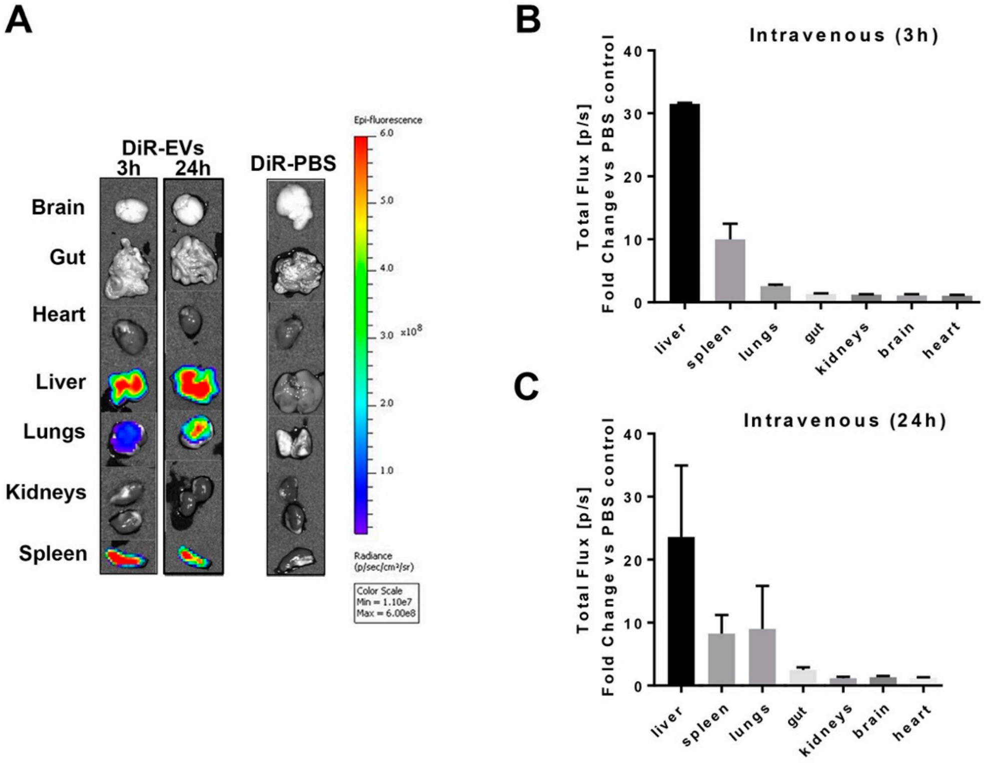

Figure S2: Analysis of DiR-labeled MSC-EV organ biodistribution. Representative image of ex vivo organ imaging using IVIS® (A). Quantification of the fluorescence intensity as total flux (photons/s) is represented in (B) after 3 h, or (C) 24 h from IV injection. Data are expressed as fold change (DiR-EVs/DiR-PBS) (n=3/each).

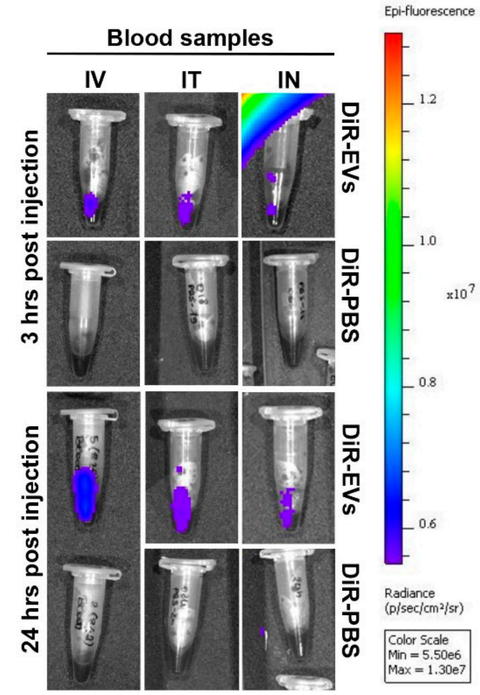

Figure S3: MSC-EV biodistribution in biofluids. Blood derived from Balb/c mice, IV, IT and IN injected with DiR-labeled MSC-EVs (DiR-EVs) or PBS (DiR-PBS). Representative analysis of the fluorescence intensity detected in blood after 3 and 24 h and measured using an IVIS® Imaging System.
